# Acquired immunity against SARS‐CoV‐2 infection and vaccination

**DOI:** 10.15252/emmm.202216345

**Published:** 2023-11-15

**Authors:** Laurent Renia, Lisa FP Ng

**Affiliations:** ^1^ Lee Kong Chian School of Medicine Nanyang Technological University Singapore Singapore; ^2^ A*STAR Infectious Diseases Labs, Agency for Science, Technology and Research (A*STAR) Singapore Singapore; ^3^ Department of Biochemistry, Yong Loo Lin School of Medicine National University of Singapore Singapore Singapore

**Keywords:** Immunology, Microbiology, Virology & Host Pathogen Interaction

## Abstract

The ongoing severe acute respiratory syndrome coronavirus 2 (SARS‐CoV‐2) pandemic has caused more than 700 million confirmed infections and ~7 million fatalities worldwide since its emergence in December 2019. SARS‐CoV‐2 is part of a family of positive‐sense, enveloped RNA viruses known as coronaviruses. Today, at least seven human coronaviruses have been identified and are known to cause respiratory tract illnesses with varying severity. The COVID‐19 pandemic spurred the generation of a vast amount of scientific knowledge on coronaviruses in record time, leading to a broad understanding of host immunity against SARS‐CoV‐2, and the rapid development of life‐saving vaccines (mainly mRNA and adenovirus‐ or inactivated virus‐based vaccines). Real world data on licensed SARS‐CoV‐2 vaccines have shown that efficacy ranges from 50 to 95% depending on viral variants, pre‐infections, and vaccine formulations, regimens, and combinations. While vaccination does markedly decrease the chances of infection and severe disease, breakthrough symptomatic and asymptomatic infections have occurred due to the emergence of immune escape virus variants. Therefore, despite these early successes, a better understanding of the mechanisms of protective immunity against infection is essential for the development of longer lasting and more efficient vaccines against SARS‐CoV‐2 and future coronaviruses.

## Graphical abstract

In this commentary, L. Renia and L. Ng discuss the humoral and cellular immune response to SARS‐CoV‐2 infection and COVID‐19 vaccination.

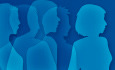

Protective immune memory, whether generated by infection or vaccination, relies on adaptive immunity that specifically targets a given pathogen. It has two main components: B cells and T cells. B cells produce antibodies that bind to the surface of the pathogen, thereby neutralising it or tagging it for destruction by other immune cells. Meanwhile, T cells recognise and kill infected cells and can also help activate other immune cells. A fraction of B cells and T cells form memory cells after infection or vaccination; these persist long after the exposure event and provide lasting protection against reinfection by the same pathogen. Numerous studies have shown that individuals who recover from mild or moderate COVID‐19 have robust and durable adaptive immunity to SARS‐CoV‐2, including neutralising antibodies, memory B cells, and memory T cells. These immune components can recognise not only the original strain of SARS‐CoV‐2 but also its variants, though the efficacy of this protection may vary depending on the degree of difference between the original and variant strains. Moreover, the adaptive immune responses against SARS‐CoV‐2 can be enhanced by vaccination, which induces higher levels of antibodies and T cells compared to natural infection. Vaccination also reduces the risk of reinfection and severe disease caused by SARS‐CoV‐2 variants.

Adaptive immunity to SARS‐CoV‐2 is a complex phenomenon that is known to vary among individuals and populations. Many factors such as viral load, early innate immunity, exposure history to SARS‐CoV‐2 or other coronaviruses, genetic background, age, comorbidities, immunosuppression, auto‐immunity, and immunodeficiency may affect the quality and extent of the adaptive immune response to COVID‐19. Individuals with an impaired immune system usually require more frequent or higher efficacy vaccination strategies to achieve substantial protection. In addition to these factors, the quality and duration of acquired immunity have also been shown to differ depending on the type of exposure – i.e., infection (naturally acquired immunity), immunisation with one or multiple vaccine types (vaccine‐induced immunity), and infection followed by vaccination or vice versa (hybrid immunity). These three types of immunity depend to different extents on the individual components of adaptive immunity.

Anti‐SARS‐CoV‐2 antibody production is triggered by the recognition of viral antigens either by antigen‐presenting cells or by B cell receptors. This leads to the activation and proliferation of SARS‐CoV‐2‐targeting B cells and T cells. B cells secrete antibodies of different classes, such as IgM, IgG, and IgA that can neutralise the virus, facilitate virus clearance by other immune cells, and/or prevent cell invasion or spike protein‐mediated cell fusion (Zohar & Alter, 2020). The spike protein covers the surface of the viral particle and is, therefore, the target for neutralising antibodies induced by infection or vaccination. The receptor‐binding domain (RBD) within the spike protein has been shown to be a primary target of these neutralising antibodies, although antibodies against the N‐terminal or the stem regions can also be neutralising. These immune features have formed the basis for the design of mRNA vaccines encoding the spike protein and for the development of therapeutic monoclonal antibodies. However, SARS‐CoV‐2 variants that display spike protein mutations in the RBD or at the N‐terminal domain can escape neutralisation, resulting in immune escape and breakthrough infections. Several studies have identified and mapped the epitopes recognised by neutralising polyclonal and monoclonal antibodies. This has helped explain the mode of actions of these antibodies, refining the development, and use of therapeutic antibodies to assess the effects of viral antigen evolution. Antibodies against other viral proteins have also been detected after infection or vaccination with inactivated viruses. Specifically, antibodies against the viral nucleoprotein have been used as a marker of infection by or exposure to SARS‐CoV‐2.

Besides recognising and/or neutralising the virus directly through their fragment antigen‐binding (Fab) domains, anti‐SARS‐CoV‐2 antibodies can also interact with immune cells (e.g., natural killer cells, monocytes/macrophages, or neutrophils) and mediators such as complement through their Fc domains. Different combinations of Fc‐mediated activities (including complement binding and activation, antibody‐dependent cytotoxicity, and antibody‐mediated phagocytosis) have been associated with different clinical outcomes (Zohar & Alter, 2020). Binding of anti‐SARS‐CoV‐2 antibodies to spike protein on viral particles or on the surface of infected cells can lead to phagocytosis by phagocytes or induce killing of infected cells through the release of cytokines and toxic components (e.g., proteases, reactive oxygen/nitrogen species). All of these pathways have been associated with the resolution of primary SARS‐CoV‐2 infection. Importantly, it has been proposed that polyclonal antibodies from vaccinated individuals that are unable to directly neutralise SARS‐CoV‐2 variants can still maintain Fc‐effector functions and limit disease severity (Bartsch *et al*, 2022).

During primary infection, neutralising anti‐spike protein IgG (mainly IgG1 and IgG3) and IgA are produced early, with IgA being the dominant virus‐neutralising isotype. However, high levels of antibodies (both IgG and IgA) have in fact been associated with disease severity, not protection (Amrun *et al*, 2020). This has been attributed to a higher viral load in the airway mucosa in patients with severe disease, leading to the continuous stimulation of specific B cells. In addition, high levels of auto‐reactive antibodies, such as antibody against type I interferons (Zhang *et al*, 2022) or specific IgG antibodies lacking fucose modification, were also associated with disease severity. The latter stimulate innate immune cells through the Fc receptor FcγRII (CD16a), leading to an inflammatory profile responsible for lung pathologies. In contrast, these pathogenic antibodies were not present in mild cases of COVID‐19.

The longevity of SARS‐CoV‐2 antibodies varies among individuals – while some people may maintain detectable levels of antibodies for several months, others may experience a rapid decline. Several factors are known to influence this. First, severity of the infection: individuals with more severe COVID‐19 infections tend to develop stronger and longer lasting antibody responses compared to milder cases. Second, vaccination: vaccination significantly boosts antibody levels in previously infected individuals and enhances the immune response. mRNA vaccines (e.g., Pfizer‐BioNTech and Moderna), and to a lesser extent adenovirus‐ or inactivated virus‐based vaccines, are known to induce robust and durable antibody responses. Third, variants of concern (VOCs): the continuous emergence of new variants has raised concerns about their potential impact on antibody responses and immunity. Some variants can partially or completely evade the antibody response generated by prior infection or vaccination, leading to a reduced effectiveness against VOCs and resulting in breakthrough infections. However, breakthrough infections have been mostly associated with mild disease. This is due in part to the increased affinity and broad repertoire of neutralising anti‐spike antibodies (Stamatatos *et al*, 2021).

The strength and breadth of the antibody response depends on the development of memory B cells. When a person is infected with the SARS‐CoV‐2 virus or is vaccinated against it, germinal centres located in the lymphoid organs produce B cells that are specific for viral components. These B cells develop via a process called affinity maturation, undergoing genetic mutations and selection to enhance their ability to effectively bind to the virus components upon re‐exposure. A subset of these B cells differentiate into plasma cells that produce large amounts of antibodies after infection or vaccination, while another subset transforms into memory B cells. The presence of memory B cells is essential for long‐term immunity to SARS‐CoV‐2. Even if antibody levels in the circulation wane over time, functional memory B cells specific for SARS‐CoV‐2 are known to persist in the bone marrow for months. This is one reason why individuals who have been infected previously or who were vaccinated against COVID‐19 tend to have milder disease or remain asymptomatic if infected/reinfected. Studies aiming to determine the longevity and effectiveness of memory B cells against the continuously occurring new variants of SARS‐CoV‐2 and how they contribute to long‐term protection against the virus are needed. They will allow the design of vaccines that trigger the generation of antibodies with greater reactivity and/or that target more conserved regions.

In addition to antibodies, T cells also play an important role in the immune response against SARS‐CoV‐2 reinfection or vaccination. T cells are divided into two subsets: CD4^+^ T cells and CD8^+^ T cells. CD4^+^ T cells (helper T cells) produce cytokines that help B cells to develop into plasma or memory subsets and to generate high‐quality antibodies, while CD8^+^ T cells (cytotoxic T cells) directly kill infected cells and prevent virus replication. T cells are induced during the first week of a primary SARS‐CoV‐2 infection in most individuals, although lymphopenia has also been observed during this time and is associated with severe disease. Conversely, a strong T cell response is associated with asymptomatic or mild disease (Bertoletti *et al*, 2022).

SARS‐CoV‐2 contains 25 different viral proteins, and T cells produced in response to natural infection recognise a large repertoire of epitopes within these proteins. These T cell epitopes are conserved in many viral variants, indicating that T cells targeting the native virus are cross‐protective against them. However, most of the widely used COVID‐19 vaccines are made of a single antigen – the spike protein. Thus, the epitope breadth of the CD4^+^ and CD8^+^ T cell responses induced by current COVID‐19 mRNA vaccines would be more restricted when compared to those induced by natural infection or by attenuated virus vaccines. However, hybrid immunity (induced by infection following vaccination or vice versa) is characterised by stronger T cell immunity with a larger breadth, and thus confers stronger immunity. It is likely that memory T cells residing in the upper airways and the lungs (tissue‐resident memory T cells or T_RM_ cells) play a strong part in this immunity since they would be able to eliminate the virus early in the infection. Patients with poor T cell responses after vaccination (e.g., elderly or immunocompromised individuals and cancer patients) would benefit from new vaccine formulations that trigger a stronger T cell immunity and are able to induce T_RM_ cells. Alternatively, T cell therapy may be an efficient approach (Bertoletti *et al*, 2022).

Some challenges and uncertainties remain regarding COVID‐19 adaptive immunity. One of these is the duration of adaptive immunity and its correlation with protection. Identifying the correlates of protection (a quantifiable marker of immune function that correlates with protection such as antibody levels against specific protein, neutralising antibody levels, etc.) is critical for informing vaccine development and better understanding the mechanisms of vaccine‐induced protection. Correlations between total and neutralising antibody levels and protection from symptomatic infections have been reported (Regev‐Yochay *et al*, 2023); however, no specific cut‐off values that define protection have been determined. In addition, these values may be applicable to the original SARS‐CoV‐2 strain (Wuhan‐Hu‐1) but not to the subsequent viral variants. Other immune parameters, such as memory B cells and T cells, have been less thoroughly investigated. Deciphering how they correlate with disease severity or with the risk of vaccine breakthrough infection will bring important knowledge. Notably, the methods for measuring the specific nature, breadth, and quality of antigen‐specific T cell responses are not easy to perform due to their inherent complexity and a lack of standardised protocols across different laboratories. However, the COVID‐19 pandemic has hastened the development of rapid, high‐throughput cellular and molecular assays that will benefit the study of adaptive immunity against SARS‐CoV‐2 and other pathogens.

Lastly, it was reported early in the pandemic that within the first group of discharged SARS‐CoV‐2‐infected patients, some individuals had sustained organ damage and were left with reduced lung functions (dubbed “long COVID”). Identification of these patients in a longitudinal cohort, together with analysis of their immune responses, would provide important and much needed knowledge for the management and treatment of these individuals (Altmann *et al*, 2023).

In conclusion, adaptive immunity is a crucial part of the immune system that provides specific and long‐lasting protection against COVID‐19. However, dysregulated adaptive responses are also associated with increased disease severity. We have gained much knowledge since the beginning of the pandemic, but gaps remain in our understanding of the duration, variability, and correlates of protection against SARS‐CoV‐2 (see Box 1). More research is needed to monitor and optimise the immune response through the development of new vaccines tailored for different settings and populations.

Box 1 **Outstanding questions on anti‐SARS‐CoV‐2 adaptive immunity.**

Which form of immunity is the most efficient at preventing infection, and how can it be harnessed to develop better vaccines?

Does immune imprinting induce by infection or vaccination impact protective efficacy against new SARS‐CoV‐2 variants or other coronaviruses?

Can cellular correlates of immunity be identified?

Can a vaccine that induces long‐lasting T_RM_ cells in the upper airways and/or lungs be developed?

Can a vaccine that completely prevents infection and transmission be developed?

Can a pan‐coronavirus vaccine be developed?

Is dysregulated adaptive immunity involved in long COVID?

## Author contributions

**Laurent Renia:** Conceptualization; formal analysis; funding acquisition; writing – original draft; writing – review and editing. **Lisa FP Ng:** Conceptualization; formal analysis; funding acquisition; writing – review and editing.

## Disclosure and competing interests statement

The authors declare that they have no conflict of interest.
